# Oxidative phosphorylation in HIV-1 infection: impacts on cellular metabolism and immune function

**DOI:** 10.3389/fimmu.2024.1360342

**Published:** 2024-03-11

**Authors:** Natalia Rodriguez Rodriguez, Trinisia Fortune, Esha Hegde, Matthew Paltiel Weinstein, Aislinn M. Keane, Jesse F. Mangold, Talia H. Swartz

**Affiliations:** Department of Medicine, Division of Infectious Diseases, Icahn School of Medicine at Mount Sinai, New York, NY, United States

**Keywords:** HIV-1, oxidative phosphorylation, antiretroviral therapy (ART), mitochondrial dysfunction, immune metabolism

## Abstract

Human Immunodeficiency Virus Type 1 (HIV-1) presents significant challenges to the immune system, predominantly characterized by CD4^+^ T cell depletion, leading to Acquired Immunodeficiency Syndrome (AIDS). Antiretroviral therapy (ART) effectively suppresses the viral load in people with HIV (PWH), leading to a state of chronic infection that is associated with inflammation. This review explores the complex relationship between oxidative phosphorylation, a crucial metabolic pathway for cellular energy production, and HIV-1, emphasizing the dual impact of HIV-1 infection and the metabolic and mitochondrial effects of ART. The review highlights how HIV-1 infection disrupts oxidative phosphorylation, promoting glycolysis and fatty acid synthesis to facilitate viral replication. ART can exacerbate metabolic dysregulation despite controlling viral replication, impacting mitochondrial DNA synthesis and enhancing reactive oxygen species production. These effects collectively contribute to significant changes in oxidative phosphorylation, influencing immune cell metabolism and function. Adenosine triphosphate (ATP) generated through oxidative phosphorylation can influence the metabolic landscape of infected cells through ATP-detected purinergic signaling and contributes to immunometabolic dysfunction. Future research should focus on identifying specific targets within this pathway and exploring the role of purinergic signaling in HIV-1 pathogenesis to enhance HIV-1 treatment modalities, addressing both viral infection and its metabolic consequences.

## Introduction

Human Immunodeficiency Virus Type 1 (HIV-1) presents a chronic, intractable challenge, primarily characterized by extensive CD4^+^ T cell depletion (1–5). This pathology manifests in both lymphoid tissues and peripheral blood, culminating in profound immunodeficiency and progression to Acquired Immunodeficiency Syndrome (AIDS). Despite the efficacy of Antiretroviral Therapy (ART) in viral load suppression, HIV-1 infection is associated with chronic inflammation and, together with the direct effects of ART, is associated with metabolic dysregulation, enhanced inflammatory response, gene expression modulation, and biochemical pathway alterations (6–9).

We explore the confluence of oxidative phosphorylation and cellular metabolic transformations in the context of HIV-1 infection and ART in PWH. Key focus areas include the nuances of mitochondrial dysfunction, specifically alterations in oxidative phosphorylation and association with immune cell dysregulation. This underscores a potential nexus between mitochondrial functionality and immunological response. Additionally, we examine the long-term impacts of ART and the potential for more nuanced HIV-1 treatment modalities. This review aims to enrich the understanding of the intricate interplay between oxidative phosphorylation and HIV-1 pathogenesis, steering future research and therapeutic interventions in this critical domain.

## Oxidative phosphorylation: an overview

Oxidative phosphorylation, a crucial biochemical process, involves the reduction of oxygen to generate ATP (10). Oxidative phosphorylation is the final stage of aerobic respiration, following glycolysis and the citric acid cycle. The efficiency of oxidative phosphorylation relies on successive oxidative/reductive reactions, notably the transfer of electrons by NADH and FADH2 to oxygen, the ultimate electron acceptor (11). The electron transport chain (ETC) facilitates this electron movement within mitochondria. In order of oxidative/reductive reactions, the complexes are I, II, coenzyme Q, III, cytochrome C, and IV. This process pumps protons across the mitochondrial intermembrane space, generating a proton electrochemical gradient that powers ATP synthase (complex V) (12). ATP synthase facilitates ATP biosynthesis with its rotating F0 and F1 components. The F1 component binds nucleotides at its catalytic sites, occupied by Mg-ADP and phosphate. Rotation, driven by F0 subunit reionization, alters F1 directionality, initiating ATP synthesis from ADP and phosphite (13). ATP generated through oxidative phosphorylation, the final and most efficient stage of aerobic respiration in the electron transport chain, serves as the primary energy source for cells, far surpassing the yields from glycolysis and the citric acid cycle.

The regulation of oxidative phosphorylation is complex and multifaceted. The mitochondrial membrane potential is at the center of the regulation, formed by the proton gradient and linked to both ATP and free radical production (14, 15). The most basic regulation of oxidative phosphorylation is allosteric control by negative feedback of substrates or intermediaries. A high NADH/NAD+ ratio slows the Krebs cycle, ETC, and oxidative phosphorylation, as does a high ATP/ADP ratio, in which ATP binds directly and inhibits cytochrome c (Cyt-c) and cytochrome oxidase (complex IV) (15, 16).

Oxidative phosphorylation complexes and Cyt-c are targeted for phosphorylation by regulatory kinases, including protein kinase C, cAMP-dependent tyrosine kinases, and EGFR (17–19). The formation of super-complexes (SCs) or respirasomes, consisting of various combinations of the ETC complexes, increases respiratory efficiency and decreases ROS production. SC abundance, along with expression of specific isoforms of Cyt-c or cytochrome oxidase, allow for regulation of ROS formation and energetic needs at tissue-level specificity (15). The fusion and fission of the mitochondrial network contribute to oxidative phosphorylation efficiency regulation, with highly connected networks promoting efficiency and curbing excess ROS production (20, 21).

## HIV-1 infection, metabolic preprogramming, cellular metabolism, and oxidative phosphorylation

Metabolic reprogramming is key in both cancer and HIV-1 infections. In cancer, this is seen as the Warburg effect, characterized by increased glucose uptake and lactate production, even when oxygen is available (22). Similarly, HIV-1 uses metabolic reprogramming to gather free nucleotides, amino acids, and lipids for viral replication and assembly (23). Studies reveal that CD4+ T cells with higher oxidative phosphorylation and glycolysis are more prone to HIV-1 infection, with infected cells showing elevated metabolic activity (24). HIV-1 also boosts glycolysis in CD4+ T cells by upregulating GLUT1 expression. The HIV-1 glycoprotein gp120 is implicated in this process, possibly by activating surface signaling molecules like CXCR4 and CCR5 and increasing the expression of glycolytic enzymes (25). Additionally, hexokinase activity is heightened in HIV-1 infected CD4+ T cells, a change dependent on viral replication (26, 27). However, the precise viral mechanisms promoting glycolysis upregulation by HIV-1 are not fully understood.

There is growing interest in understanding the impact of HIV on Mitochondrial-associated ER membranes (MAMs) due to their crucial role in metabolic reprogramming. MAMs serve as structures linking the endoplasmic reticulum (ER) and mitochondria, facilitating oxidative protein production and mitochondrial biogenesis. The viral HIV-1 Tat protein disrupts MAMs in neuronal cell lines upon infection by phosphorylating the mitochondrial protein PTPIP51 and disrupting its localization to MAMs, thus reducing calcium signaling and increasing ROS accumulation (28). Furthermore, HIV-1 Vpr has been shown to disrupt MAMs by decreasing the expression of critical MAM-associated proteins Mfn2 and Drp1 (29). The targeting of MAMs by HIV-1 induces oxidative stress that contributes to HIV-associated metabolic reprogramming, but further research is needed to establish other key mechanisms involved.

HIV-1 impacts host cell metabolism, initially enhancing glycolysis and fatty acid synthesis for viral replication (30–33). This increased metabolic activity, particularly in glycolysis and glucose transport, makes cells like T-cells and monocytes more susceptible to HIV-1 infection (24, 34, 35). Upregulation of the GLUT-1 transporter and mitochondrial oxidative damage, linked to ROS production, highlights the role of metabolic reprogramming in HIV-1 infection and potential research avenues (31, 35, 36). People with HIV (PWH) face systemic metabolic challenges due to both HIV-1 and antiretroviral therapy (ART), increasing metabolic disease risks (37, 38). Studies have shown that HIV-1 influences metabolic aging, with oxidative phosphorylation and pyruvate metabolism downregulation noted in PWH (39). Furthermore, HIV-1 affects the expression of electron transport chain (ETC) components and causes mitochondrial damage, as seen in the upregulation of Complex-IV subunit, contributing to oxidative stress (40, 41).

ART classes have long been associated with mitochondria dysfunction (42). Both Nucleoside/Nucleotide Reverse Transcriptase Inhibitors (NRTIs) and Non-nucleotide Reverse Transcriptase Inhibitors (NNRTIs) are associated with dyslipidemia in PWH and alter adipocyte differentiation. NRTIs induce lipodystrophy by promoting mitochondrial dysfunction and adipocyte death and interfering with mitochondrial DNA (mtDNA) synthesis (42). This inhibits Pol-gamma, increases ROS production, and reduces ETC activity and oxidative phosphorylation (37, 42–46). Additionally, protease inhibitors (PIs) target GLUT4, impairing glucose uptake into adipocytes and promoting insulin resistance (37, 47, 48).

HIV-1 infection exacerbates oxidative stress and mitochondrial damage, affecting cell metabolism and promoting diseases in PWH, especially those on ART. Gp120 upregulates CYP2E1, proline oxidase (POX), NOX2, and NOX4 to enhance ROS production (49–57). Nef interacts with the NADPH oxidases without affecting the NOX expression (57). The HIV-1 proteins Vpr and Tat both contribute to mitochondrial dysfunction: Vpr reduces membrane potential and triggers apoptosis by binding to ANT, part of the mitochondrial permeability transition pore, while Tat elevates free calcium levels in the cytoplasm by interacting with NADPH oxidases, leading to increased mitochondrial calcium uptake and reactive oxygen species (ROS) production (54, 55, 58–64). These changes increase the risk of metabolic diseases in PWH, with ART compounds like NRTIs and PIs exacerbating (65, 66).

## Purinergic receptors, ATP, and HIV pathogenesis

Purinergic receptors, particularly the P1 and P2 subtypes, are integral to inflammatory responses and the progression of HIV-1. The P2X receptors, activated by ATP, are crucial in initiating immune responses and impacting the HIV-1 viral life cycle (67–71). The enzyme CD39 regulates ATP availability, influencing both the progression of AIDS and the function of T cells by modulating ATP and adenosine levels, affecting P2X receptor signaling (72–74).

The P2X receptors are implicated in cell entry and infection by the virus, and their inhibition can significantly reduce HIV-1 infection in various cell types (70, 75–85). Chronic inflammation in HIV is driven by factors like immunosenescence and persistent viral replication, even with antiretroviral therapy (ART). It is exacerbated by factors such as organ fibrosis and co-infections (4, 9, 86–88).

Furthermore, extracellular ATP and its interaction with P2X receptors regulate HIV infection and inflammation. This signaling leads to the production of inflammatory cytokines and the activation of the NLRP3 inflammasome, contributing to CD4+ T lymphocyte depletion and apoptosis (77, 89–92). HIV-1 interacts with Pannexin-1, a membrane channel, suggesting a link between ATP production and inflammatory signaling in HIV-1 infection (93–96). Circulating levels of ATP have been proposed as a biomarker of cognitive decline in PWH, predicting central nervous system compromise and suggesting the use of Pannexin-1 or purinergic receptor inhibitors for clinical intervention (97).

## HIV-associated neurocognitive disorder: the roles of HIV-1, ART, oxidative phosphorylation, and purinergic signaling

The progression of HIV-1-associated neurocognitive disorders (HAND) in PWH has been linked to purinergic signaling and oxidative stress. The central nervous system serves as a reservoir of HIV-1, leading to neuroinflammation and neurodegeneration that progress to HIV-1 encephalitis and HAND that persist despite the use of effective ART (98–100). Within the CNS, HIV-1 induces the release of extracellular ATP in infected cells to support viral replication, and ATP interacts with purinergic receptors to produce a proinflammatory response (83, 101, 102). The purinergic receptor subtype P2X is particularly interesting, notably P2X7, which is widely expressed in various brain cells, including astrocytes, oligodendrocytes, microglia, and neurons (102). In astrocytes, P2X7 activation induced by gp120 leads to Cx43 hemichannels and pannexin-1 opening, resulting in increased ATP release and nitric oxide production. This process is speculated to propagate gp120-mediated signaling to neighboring cells (95). In the peripheral nervous system (PNS), P2X4 is involved in gp120-induced lysosomal exocytosis and ATP release in Schwann Cells, increasing cytosolic calcium and generating ROS in dorsal root ganglia neurons (101). Although ART mitigates the neuropathology of HIV-1 infection in PWH, it does not eliminate it, with studies showing possible neurotoxicity of ART. ART-related neurotoxicity in the brain is thought to be linked to the loss of mtDNA due to NRTIs inhibiting mtDNA polymerase gamma (103, 104). Additionally, ART contributes to astrocyte autophagy, de-acidification of endolysosomes, and the promotion of amyloidogenesis, all associated with the pathogenesis of HAND (105).

Extracellular ATP and P2X receptors are integral in HIV-1 infection, influencing the virus’s life cycle and contributing to immunopathogenesis, neurodegeneration, and chronic inflammation. Their interaction with HIV-1 is key in disease progression, affecting viral replication, cytokine release, and cell death (75, 77, 78, 81, 83–85). This insight reveals the potential of targeting purinergic signaling in developing novel therapies for HIV-1, reducing inflammation and related comorbidities, and represents a significant area for future research.

## Oxidative phosphorylation in immune cell dysfunction during HIV-1 infection

The metabolic dynamics of immune cells, particularly CD4+ T cells, CD8+ T cells, and macrophages, are critical in understanding HIV-1 pathophysiology. CD4+ T cells infected with HIV-1 shift towards increased glycolysis, facilitating viral replication. A study identified a link between oxidative phosphorylation in these cells and higher viral loads, with NLRX1 and FASTKD5 as key factors. Metformin, a complex I inhibitor, was found to suppress HIV-1 replication in CD4+ T cells (106). In PWH on ART, there’s an upregulation of oxidative phosphorylation compared to elite controllers, and complex IV inhibition was linked to increased HIV-1 reactivation in a latency model (107).

CD8+ T cells in chronic HIV-1 infection experience metabolic exhaustion, diminishing their functionality and virus control ability. A combination therapy comprised of a mitochondrial superoxide scavenger, a small-molecule inhibitor of mitochondrial fission, and IL-15 can increase the frequency of IFNy and TNFa poly-functional CD8+ T cells and decrease the frequency of exhaustion markers (108).

In HIV-infected macrophages, there is notable support for prolonged HIV-1 replication and survival, shielding virions from ART and neutralizing antibodies. These macrophages show metabolic plasticity, shifting between oxidative phosphorylation and glycolysis based on their polarization state. HIV-1 infection alters macrophage metabolism, promoting a pro-inflammatory state and increasing glycolytic activity, which is linked to chronic inflammation seen in HIV-1 infection (109). A study demonstrated that superoxide dismutase (SOD) mimetic drugs could inhibit myeloid cell-driven bystander cell death *in vitro* (110).

**Figure 1:**
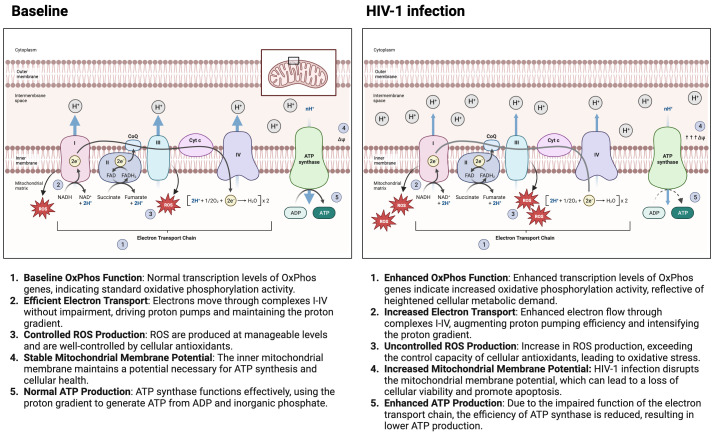
HIV-1 Infection impact on mitochondrial oxidative phosphorylation. The figure contrasts mitochondrial function under normal conditions with the altered state during HIV-1 infection. It shows that HIV-1 infection leads to increased transcription of oxidative phosphorylation genes, higher electron transport efficiency, and elevated reactive oxygen species (ROS) production, resulting in an enhanced mitochondrial membrane potential and increased ATP production compared to the baseline state. These changes indicate a hyperactivated mitochondrial state in response to HIV-1 infection. Made with biorender.com.

**Figure 1** highlights the impact of HIV-1 infection on mitochondrial oxidative phosphorylation. It contrasts the normal mitochondrial function with the altered state during HIV-1 infection, depicting how HIV-1 infection leads to an upregulation in the transcription of oxidative phosphorylation genes, resulting in greater electron transport chain efficiency. This heightened activity leads to increased production of ROS, a hallmark of cellular stress. Consequently, there’s an enhancement in mitochondrial membrane potential and a notable increase in ATP production compared to baseline conditions. These alterations signify a hyperactivated mitochondrial state triggered by HIV-1 infection, underlining the complex interplay between the virus and the host’s cellular metabolism.

## Current research and future directions

Therapeutic interventions targeting mitochondrial dysfunction and oxidative stress in HIV immunopathogenesis are emerging as a crucial field of research. Metformin, a diabetes medication, has shown varying effects on HIV-1 replication in studies by Rezai et al. and Guo et al. (106, 111). There is an additive effect of risk of metformin-associated lactic acidosis when used with NRTIs like tenofovir, which calls for caution in its clinical application (112, 113). Research on the antioxidant properties of plant flavonoids, such as naringin, is gaining attention for their potential to mitigate oxidative damage induced by NRTIs (114, 115). Statins, known for their anti-inflammatory and lipid-lowering effects, are being explored for their benefits in HIV management, with studies like SATURN-HIV and REPRIEVE indicating their impact on cardiovascular risk and mitochondrial function in PWH on ART (116–118).

Given that HIV-1 infection induces metabolic reprogramming like that observed in cancer cells, there is growing interest in exploring anti-cancer drug treatments as potential interventions to target these metabolic effects of HIV-1 (119). In neurons, the viral protein gp120 reduces ATP output via oxidative phosphorylation while increasing glycolysis production and PKM2 expression. Tepp-46, a selective PKM2 tetrameric stabilizer and a potential small molecule therapeutics for lung cancer, has demonstrated the ability to reverse the metabolic reprogramming induced by gp120 in neuronal cells (120). Further, studies have linked dyslipidemia in PWH on ART with mitochondrial oxidative stress, suggesting an association between lipid profiles and the function of mitochondrial electron transport chain complexes (121). Atovaquone, a mitochondrial complex III inhibitor, though not tested in chronic inflammation, is used for treating parasitic and fungal infections (81).

Advancements in understanding HIV-1 pathogenesis highlight the role of purinergic signaling, particularly the involvement of extracellular ATP and P2X receptors. These receptors play a significant role in the inflammatory response and immune modulation in HIV-1 infection (112, 113). This growing body of research underlines the importance of understanding the metabolic and mitochondrial aspects of HIV-1 infection and ART. The goal is to improve treatment outcomes by mitigating adverse effects and exploring new therapeutic avenues that address both HIV-1 management and broader metabolic and cardiovascular health.

## Conclusions

The interaction between HIV-1 infection and metabolic activity significantly shapes the chronic inflammation commonly associated with HIV. The activation of oxidative phosphorylation is a key driver in chronic inflammation. This shift is evident in studies documenting the increased activity of mitochondrial respiratory chain complexes in HIV-1 infected cells (122–132). Such changes suggest that HIV-1 exploits the host’s mitochondrial machinery to its advantage, potentially exacerbating the inflammatory response. While ART effectively controls viral replication, it does not fully rectify this metabolic dysregulation, adding another layer to the complexity. Long-term ART use has been associated with continued mitochondrial dysfunction (133, 134), indicating that the impact of HIV on oxidative phosphorylation is a persistent challenge.

HIV-1 infection significantly disrupts host cell metabolism, increasing glycolysis and fatty acid synthesis to enhance viral replication, leading to oxidative stress and mitochondrial damage. This is further complicated by antiretroviral therapy (ART), which contributes to metabolic conditions like dyslipidemia and insulin resistance, intensifying mitochondrial dysfunction (135–137).

Studies show that both HIV-1 infection and prolonged ART use increase the risk of oxidative phosphorylation dysregulation and mitochondrial disruption in people with HIV (PWH) (122–132). Reduced activity of respiratory chain complexes and altered mitochondrial functioning in ART-naïve PWH have been linked to neurocognitive impairment (125). ART’s impact on oxidative phosphorylation in PWH also suggests a reduction in mitochondrial function associated with chronic/controlled HIV-1 (133). Interestingly, pre-exposure prophylaxis (PreP) also shows reduced mitochondrial function in healthy individuals (134). Gender-specific differences in response to long-term ART have been observed, indicating possible variations in immunological recovery (138).

Recent studies highlight the role of purinergic signaling, particularly the interaction between extracellular ATP and P2X receptors, in HIV-1 pathogenesis (112, 113). These receptors, activated by ATP released from stressed cells, influence the HIV-1 life cycle and contribute to immunometabolic dysfunction (96, 106, 109). The dual role of these receptors in potentially inhibiting or facilitating HIV replication is mediated through CD39, which modulates extracellular ATP levels (72, 139–142).

Understanding the interplay between HIV-1 infection, ART, oxidative phosphorylation, and purinergic signaling is crucial for developing comprehensive HIV-1 treatment strategies. This involves addressing the viral challenges and the broader metabolic and mitochondrial dysfunctions. Future research should focus on identifying specific targets influenced by HIV-1 and ART and exploring the impact of purinergic signaling on these pathways. Such targeted exploration may lead to innovative therapeutic approaches addressing the infection and its metabolic and mitochondrial consequences.

## Author contributions

NR: Conceptualization, Data curation, Investigation, Methodology, Project administration, Software, Supervision, Validation, Visualization, Writing – original draft, Writing – review & editing. TF: Writing – original draft. EH: Writing – original draft. MW: Writing – original draft. AK: Writing – original draft. JM: Writing – original draft. TS: Conceptualization, Data curation, Formal Analysis, Funding acquisition, Investigation, Methodology, Project administration, Resources, Software, Supervision, Validation, Visualization, Writing – original draft, Writing – review & editing.
